# Amorphous Carbon Nitride Films: Surface and Subsurface Composition and Bonding

**DOI:** 10.1021/acs.langmuir.4c02007

**Published:** 2024-08-30

**Authors:** Josef Zemek, Jana Houdkova, Petr Jiricek, Tomas Kocourek

**Affiliations:** a Institute of Physics of the Czech Academy of Sciences, Na Slovance 2, 182 21 Prague 8, Czech Republic; b Czech Technical University in Prague, Faculty of Biomedical Engineering, nam. Sitna 3105, 27201 Kladno, Czech Republic

## Abstract

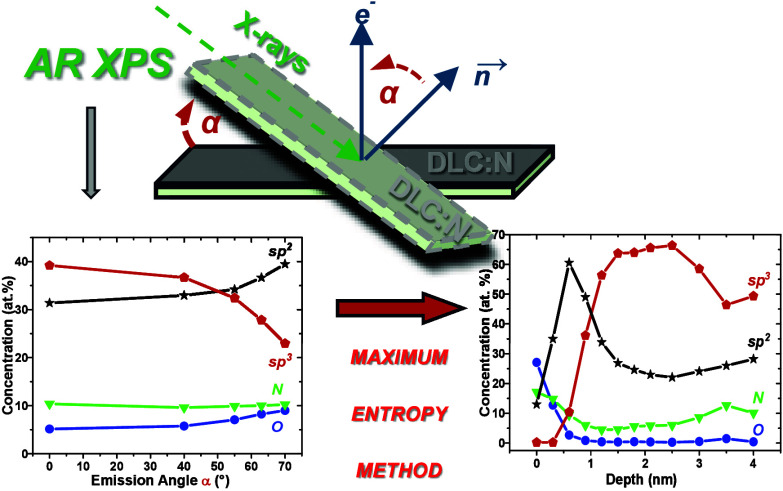

To obtain quantitative information about the composition and bonding of atoms located at and beyond the analyzed solid surface nondestructively, we applied angle-resolved X-ray photoelectron spectroscopy aided by the maximum entropy method to air-exposed amorphous carbon nitride films deposited by pulsed laser deposition of diamond-like carbon modified by low-energy nitrogen ion bombardment during film growth. We demonstrate that the composition, chemical bonding, and mass density vary significantly from the top surface to a shallow subsurface region. The analyzed samples, in a shallow surface region of ∼1 nm, are composed of oxygen, nitrogen, hydrogen, and mostly carbon in sp^2^ hybridization. In a deeper region, the C sp^3^ content increases substantially going to a maximum, whereas the nitrogen percentage decreases to a minimum, then increases, and tends to saturate. Special attention has been paid to in-depth distributions of carbon atoms in trigonal and tetragonal arrangements because they specify numerous physical and chemical properties of carbon-based materials. These results indicate that the interaction of DLC:N surfaces with surroundings can be influenced, barring oxygen and nitrogen, by sp^2^-bonded carbon atoms located near the surface of the samples. The obtained results can be useful for developing a deeper understanding of the interaction between DLC:N layer surfaces and their surroundings and particularly with living tissue.

## Introduction

In material research of solids, various surface- and bulk-sensitive analytical methods supply valuable information about the composition and chemical bonding of elements. There is, however, no straightforward way to obtain this information in a shallow near surface region of analyzed samples. In fact, atoms located on the top surface and in a shallow subsurface region and their chemical bonds play an important role in properly understanding surface chemistry in the interaction with surroundings, particularly at the solid–liquid and solid–gas boundaries.

In surface analysis, soft X-ray photoelectron spectroscopy (XPS) and Auger electron spectroscopy (AES) provide useful information about the composition and bonding averaged within the information depth (ID) of the photoelectrons. The ID is approximated by 3λ cos α, where λ is the inelastic mean free path (IMFP) of electrons in question and α is the electron emission angle measured from the surface normal.^1^ The ID, as defined above, represents the thickness of a surface layer from which 95% of the spectral intensity can be recorded, typically 5–10 nm, assuming a laboratory radiation source, Al Kα, at 1487 eV. Evidently, the ID (and therefore the surface sensitivity) changes extensively with the electron emission angle. A set of measurements at different emission angles exploits the natural variation of the ID. This effect can be utilized to obtain qualitative information about the surface and near surface composition and chemistry.^2,3^ Similar qualitative information can also be obtained by comparing electron spectra with rather different kinetic energies of signal electrons.^4^ An alternative method is sputter depth profiling, which uses energetic ion beams to remove materials from a surface. Sputter depth profiling is, however, a destructive method and can produce artifacts, including atom mixing at the sputtered surface, preferential sputtering of some of the specimen components, and implantation of primary beam ions.^5^

The in-depth distribution of elements and bonding states in surface regions of carbon nitride films is rare and limited to qualitative information. Hellgren et al.^6^ have studied magnetron-sputtered CN_*x*_ films, without air exposure and after air exposure and finally after Ar ion etching at ion energies ranging from 500 eV to 4 keV, by angle-resolved XPS (ARXPS). They revealed a surface enrichment of nitrogen and a preferential sputtering of nitrogen following sputter cleaning. The first attempt (and likely the only one) to convert ARXPS spectra into concentration depth profiles of carbon nitride films prepared by in situ low-energy (1–5 keV) nitrogen ion irradiation of graphite and diamond was published as early as in 1997.^7^ The nitrogen in-depth distribution forms roughly Gaussian peak shapes with, curiously, nearly independent maximum positions on the ion energy.

ARXPS aided by the maximum entropy method (MEM) may overcome the difficulties mentioned above, providing a chance to obtain nondestructive reconstructions of the compositional depth profile of a solid surface within the ID. This requires, however, an inversion of the Laplace transform that does not have a unique solution, and it is strongly influenced by the noise of the spectra. To overcome the problem, it is necessary to use regularization.^8^

The MEM, first introduced by Smith and Livesey, is one of the numerically stable methods used to recover compositional and chemical bonding depth profiles from ARXPS data.^8,9^ The software used in this work^10^ has been successfully tested^11^ and has also been applied to various measured data.^11−16^ Szklarczyk et al.^13^ have confirmed that real spectroscopic data, recorded for a self-assembling layer containing iron and nitrogen, can be reconstructed with a subnanometer depth resolution of 0.2 nm. ARXPS with the MEM has also been successfully used to recover compositional and bonding depth profiles of a single-layer graphene on copper^15^ and has confirmed the excellent depth resolution of the method near the top surface of the sample. The fractional depth resolution depends primarily on the percentage precision of the individual peak intensity measurements.^17^

In this work, we continue to conduct material research of carbon-based materials, namely, by using ARXPS. Most of the published works on this topic examine various physical properties of DLC films linking them to carbon atom hybridizations. There is, however, a problem with the interpretation of the obtained hybridization content. The C sp^2^ and C sp^3^ contributions are currently derived from the photoelectron C 1s lines. The values are averaged within information depths of the method used under the assumption of the homogeneous distribution of elements. As we have shown recently for undoped DLC films^14^ and Ca-doped DLC films,^16^ such an assumption is questionable and can lead to confusing conclusions.

We apply ARXPS with the MEM primarily on air-exposed nitrogen-doped diamond-like carbon (DLC:N) layers grown on silicon wafers. Such layer surfaces are amorphous and adequately smooth and contain a limited number of elements: carbon, nitrogen, and oxygen. In addition, electron elastic scattering in the analyzed material is weak and can therefore be neglected. However, measured photoelectron spectral signals also depend on the mass density in a near surface region of the samples.^1,17^ Therefore, to approximate the mass density, we recorded the reflection electron energy loss spectra (REELS) at several primary electron beam energies and therefore at different IDs. All of the material properties mentioned above are eligible for successful in-depth reconstruction.^17^ Differentiation of chemical bonds of atoms located at a surface region of the samples is, however, a more complex problem. This is due to multiple bonding states of carbon, nitrogen, and oxygen atoms and their spectral overlap, and finally, this is still an active field of research at least for nitrogen.^18−23^

## Materials and Methods

### Samples

Diamond-like carbon (DLC) and DLC:N films were grown in a hybrid deposition setup consisting of pulsed laser deposition (PLD) and a model eH200 ion source (Kaufman and Robinson, Inc.).^24^ A KrF excimer laser (248 nm, pulse duration of 20 ns, 10 J cm^–2^, 1800 pulses) was used for the ablation of graphite target in a low-pressure molecular nitrogen atmosphere, whereas the ion source was used to modify the growing film by nitrogen ion beam bombardment (mostly N_2_^+^) at low ion energies of 40–70 eV. Substrates from Si(100) wafers were held at room temperature during deposition. The thickness of the layers reached ∼100 nm. Before analysis, the samples were kept in air under laboratory conditions for approximately one year. The deposition parameters of ion beam irradiation during film growth are summarized in Table 1.

**Table 1:** Parameters of the Nitrogen Ion Beam and Nitrogen Working Pressure during DLC:N Film Growth^a^

sample	ion beam energy (eV)	ion beam current (A)	N_2_ pressure (Pa)
B0	–	–	–
B1	40	0.135	0.40
B2	50	0.145	0.15
B3	70	0.140	0.40

a Note that sample B0 is a nitrogen free DLC reference film.

### Spectrometers

ARXPS spectra were recorded with an AXIS-Supra photoelectron spectrometer (Kratos Analytical Ltd.), using monochromatized Al Kα radiation (1487 eV, 300 W, analyzed area of 0.7 mm × 0.3 mm). Air-exposed samples did not undergo any surface cleaning treatment before they were introduced into the spectrometer chamber. The high-energy resolved C 1s, N 1s, and O 1s spectra were recorded with a pass energy of 10 eV, with a step of 0.1 eV, resulting in an overall energy resolution of 0.45 eV. Binding energy calibration was performed with respect to the C sp^2^ contribution of the fitted C 1s lines that peaked at 284.3 eV. The angle-resolved spectra were recorded by tilting the samples at recommended emission angles of 0°, 40°, 55°, 63°, and 70° measured from the surface normal,^17^ with an acceptance angle of ±4°. Quantification was performed using the integrated peak areas of the C 1s, O 1s, and N 1s core level spectra after standard Shirley’s electron inelastic background subtraction and using the atomic sensitivity factors given in ESCApe software (Kratos Analytical Ltd.). The C 1s spectra were analyzed by peak fitting using the asymmetric pseudo-Voigt peak shape to separate the C sp^2^ bonds,^25^ and the symmetric Voigt curves were used to separate the remaining contributions. Angle-dependent apparent concentrations were used as input data for depth profile reconstructions using the MEM, provided by Kratos Analytical Ltd.

REELS spectra were recorded using an angle-resolved photoelectron spectrometer (ADES 400, VG Scientific UK) equipped with an electron gun (Kimbal Physics Inc., EGPS-3101E). Electron loss spectra were induced by the primary electron beam passing along the surface normal with a spot diameter of 3 mm at the sample surface. The electron analyzer was set at 35° from the surface normal at a pass energy of 20 eV. The REELS spectra were excited with electron beam energies of 0.5, 1.0, and 2.0 keV. The spectra were used to estimate the mass density and to detect hydrogen (occurring in expected OH, CH_*x*_, and H_2_O bonding states) in the surface region of the samples.

After the analysis of air-exposed surfaces of the samples, the surfaces were sputtered for 15 min (B0–B2) or 60 min (B3) (denoted sputter-cleaned surfaces) by an argon cluster ion beam (ArCIB) to remove surface contamination and then analyzed by XPS. The ion beam impact area was 2 mm × 2 mm at an incident angle of 50° from the surface normal. The following sputtering conditions were adjusted and were used in this work: 5 keV primary ion beam energy and 7 nA primary ion beam current. The average number of Ar atoms in the clusters was set to 2000. The average energy per Ar atom in clusters was as low as 2.5 eV. Sputter cleaning under the conditions used is expected to have a very gentle impact on the treated surfaces. The mean sputtering yield, determined by atomic force microscopy (AFM) from the crater sputtered for an extended period of time, was rather low, 0.13 nm/min.^14^

## Results and Discussion

This section is organized into four subsections. The first two subsections address the surface composition and photoelectron spectra of air-exposed and sputter-cleaned DLC:N films recorded using common XPS. In the third subsection, qualitative in-depth information is gained from the ARXPS spectra. Finally, in the fourth, the mass density values and nondestructive concentration depth profiles are presented and discussed.

### Surface Composition of Air-Exposed and Sputter-Cleaned Surfaces

Only C, O, and N peaks are observed in the XPS survey spectra (Figure S1). Apparent atomic composition values calculated from C 1s, N 1s, and O 1s spectra recorded at the normal electron emission angle from the air-exposed and sputter-cleaned surfaces of the analyzed samples are summarized in Table 2.

**Table 2:** Apparent Atomic Concentrations of Elements Found in a Surface Region of the Analyzed Samples, Calculated from C 1s, N 1s, and O 1s Peak Areas Recorded at the Normal Electron Emission Angle, Assuming a Homogeneous Distribution of Elements in a Surface Region of the Samples^a^

sample	sample surface	ion energy (eV)	C (atom %)	N (atom %)	O (atom %)	C sp^2^ (%)	C sp^3^ (%)	C sp^3^/C sp^2^
B0	air-exposed	0	96.0	–	4.0	24.8	66.9	2.70
	sputter-cleaned	0	99.3	–	0.7	28.0	72.0	2.57
B1	air-exposed	40	85.8	8.8	5.4	37.6	34.0	0.90
	sputter-cleaned	40	93.4	5.8	0.7	54.4	45.6	0.84
B2	air-exposed	50	90.8	4.2	5.0	42.3	39.1	0.92
	sputter-cleaned	50	94.5	4.6	0.9	47.6	52.4	0.90
B3	air-exposed	70	85.6	9.4	5.0	32.2	38.2	1.19
	sputter-cleaned	70	93.2	6.8	0.0	33.7	49.7	0.68

a The C sp^2^ and C sp^3^ percentages related to the C 1s peak areas and C sp^3^/C sp^2^ ratios are added.

Oxygen concentrations reached 4–5 atom % at the surfaces of the air-exposed samples. After mild sputter cleaning, only traces of oxygen are found (<1 atom %) and no spectral signal from Ar is observed. The nitrogen concentration reaches 4–9 atom %. The C sp^2^ percentage derived from peak fits of the C 1s lines increased from 25% for the air-exposed DLC sample to ∼40% for the air-exposed DLC:N films. For the sputter-cleaned samples, the C sp^2^ content further increases. The sp^3^/sp^2^ ratio of the air-exposed films decreases from 2.7 (DLC) to ∼1 (DLC:N) and further decreases for the sputter-cleaned surfaces.

### Photoelectron Spectra of Air-Exposed and Sputter-Cleaned Surfaces

**Figure 1:**
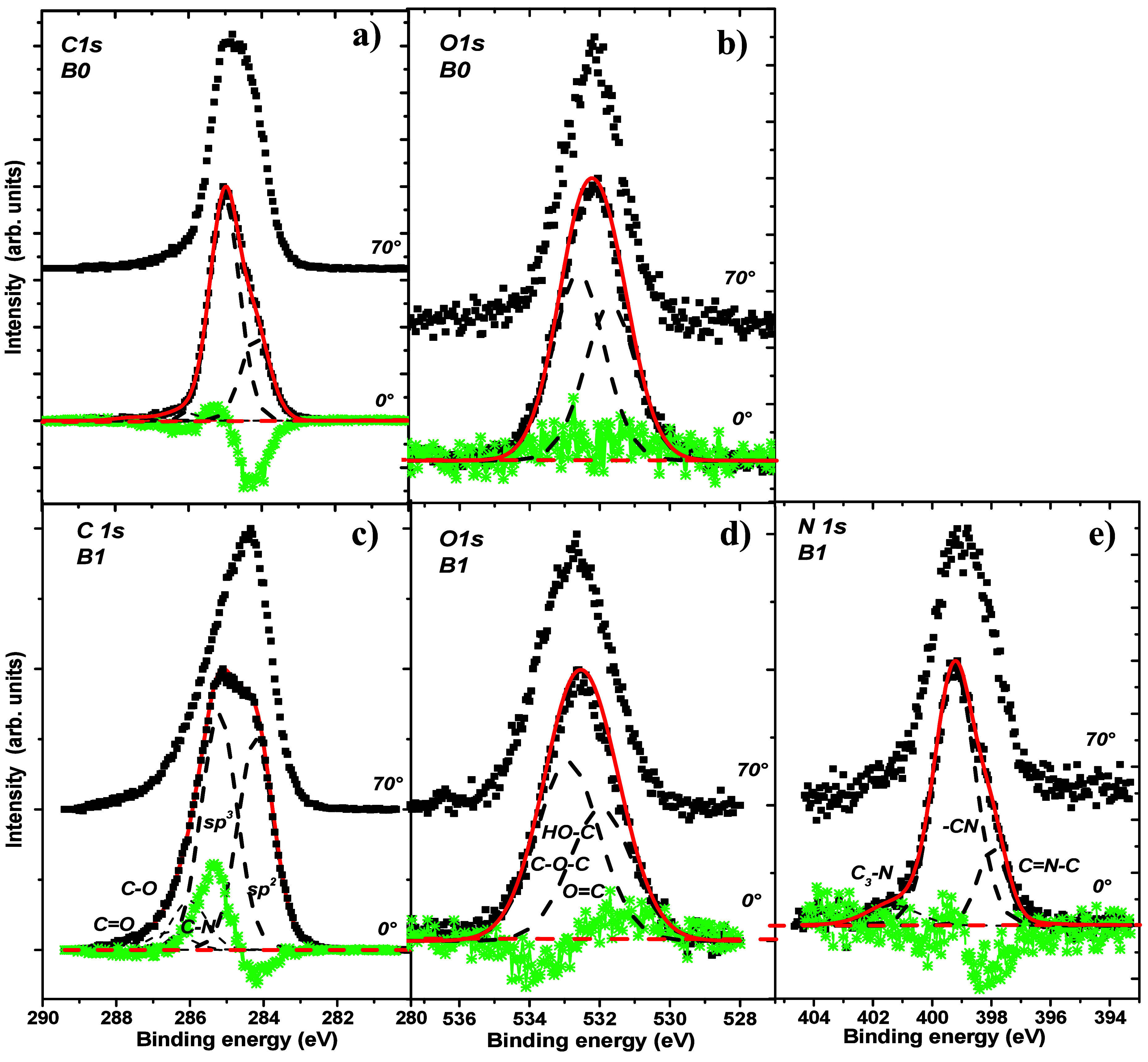
Representative high-energy resolved C 1s, O 1s, and N 1s spectra recorded at the normal 0° and inclined 70° emission angles from the air-exposed surface of (a and b) nitrogen free DLC and (c–e) DLC:N film, normalized to unity. Differential spectra (0–70°) are colored green, and peak fits are depicted with dashed lines. Note that the method is more sensitive to the surface at an inclined emission angle.

Typical high-resolution C 1s, O 1s, and N 1s spectra recorded for the DLC and DLC:N samples are shown in panels a and b and panels c–e, respectively, of Figure 1. All of the spectra are acquired at two distinct emission angles, 0° (corresponds to electron emission along the surface normal) and 70°, to emphasize the changes induced by the distinct surface sensitivity of XPS.

The C 1s line is fitted by sp^2^ and sp^3^ predominant contributions located at 284.3 and 285.1 eV, respectively, while the minor states of bonding of carbon to nitrogen (286.0 eV) and carbon to oxygen (C–O, 286.6 eV; C=O, 287.7 eV) are located at higher binding energies.^6,26^ The N 1s envelope is fitted using three sublines ascribed to pyridinic nitrogen (398.0 eV), to nitriles (399.2 eV),^18,26−28^ and/or to a polarization shift in pyridinic nitrogen^6^ and to graphite-like nitrogen (401.3 eV).^6,7,18,29^ The O 1s peak is not commonly studied in detail. It is fitted tentatively to double-bonded (terminated) oxygen at 532.0 eV and single-bonded oxygen peaked at 532.9 eV.^6^ The results presented above indicate that the introduction of nitrogen into DLC films increases the intensity of the C sp^2^ component, which can be discerned even with the naked eye. From the differential spectra, we deduce that the surface region of the samples is enriched by C sp^2^ bonds, by pyridinic nitrogen and by C–O bonds, all consistent with ref (6). Therefore, the in-depth distribution of elements and resolved bonding states vary, going from the surface to the bulk.

**Figure 2:**
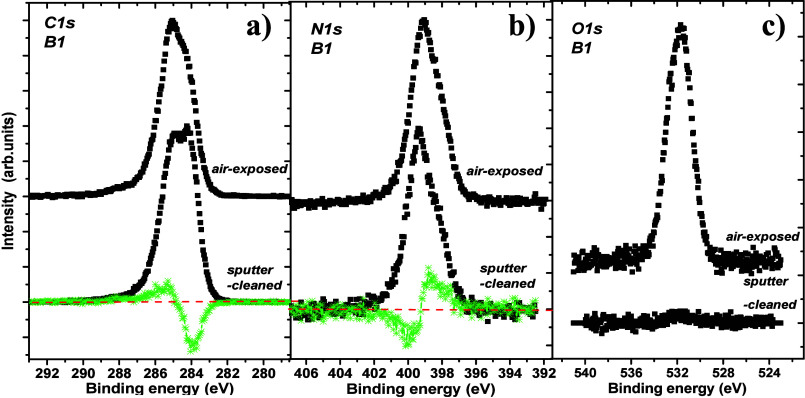
Typical high-energy resolved (a) C 1s, (b) N 1s, and (c) O 1s photoelectron spectra recorded for air-exposed and sputter-cleaned DLC:N film surfaces along the normal electron emission angle. All spectra are normalized to unity except the O 1s spectrum that was recorded for the sputter-cleaned surface.

Representative high-energy resolved C 1s, N 1s, and O 1s spectra recorded for air-exposed and sputter-cleaned DLC:N samples are shown in Figure 2a–c.

The C 1s peak maxima are located at 285.1 and 284.3 eV for the air-exposed and sputter-cleaned sample surface, respectively. Their widths [Full Width at Half-Maximum (FWHM)] reached 2.1–2.3 eV for the air-exposed surface and only 1.3 eV for the DLC surface. Obviously, this indicates additional bonding states of carbon and nitrogen atoms, a nitrogen-induced disorder present in the DLC:N samples,^30^ and a surface enrichment by C sp^2^ bonds. After sputter cleaning, the FWHM values increase only slightly by 0.1 eV, which was caused by a very mild sputter-induced disorder.^6^ N 1s spectra peak at 399.1 and 399.3 eV for the air-exposed and sputter-cleaned surface, respectively. Their FWHM values are similar, 2.0 and 2.1 eV. The O 1s spectrum recorded for the air-exposed surface peaks at 531.6 eV with a FWHM of 2.2 eV. Only traces of oxygen atoms are found on the sputter-cleaned surface, indicating a surface location of oxygen-bearing species.

These results indicate that the introduction of nitrogen into DLC films increases the intensity of the C sp^2^ component, which can be discerned even with the naked eye. From the differential spectra, we deduce that the surface region of the samples is enriched, in addition to the C sp^2^ bonds, by pyridinic nitrogen and by C–O bonds, all consistent with ref (6). Therefore, their in-depth distribution of elements and resolved bonding states vary, going from the surface to the bulk. Sputter cleaning applying the ArCIB technique to the air-exposed samples appears to be efficient in removing adventitious carbon and, particularly, oxygen-bearing species. Simultaneously, the apparent nitrogen content changes^31,32^ as evidenced in Table 2. The difference spectrum of the N 1s lines indicates the intensity decrease at ∼399 eV and the increase at ∼400 eV due to sputter cleaning. This is consistent with the mild shift of the N 1s peak maximum after sputter cleaning. The extent of structural transformation due to sputter cleaning and nitrogen introduction is discussed in Application of the MEM Approach.

### Qualitative In-Depth Information

Rough estimation of depth distributions can be done qualitatively, by examining peak areas or apparent concentrations versus electron emission angle, as presented below, and on a quantitative level by applying ARXPS with the MEM, as shown in Application of the MEM Approach.

**Figure 3:**
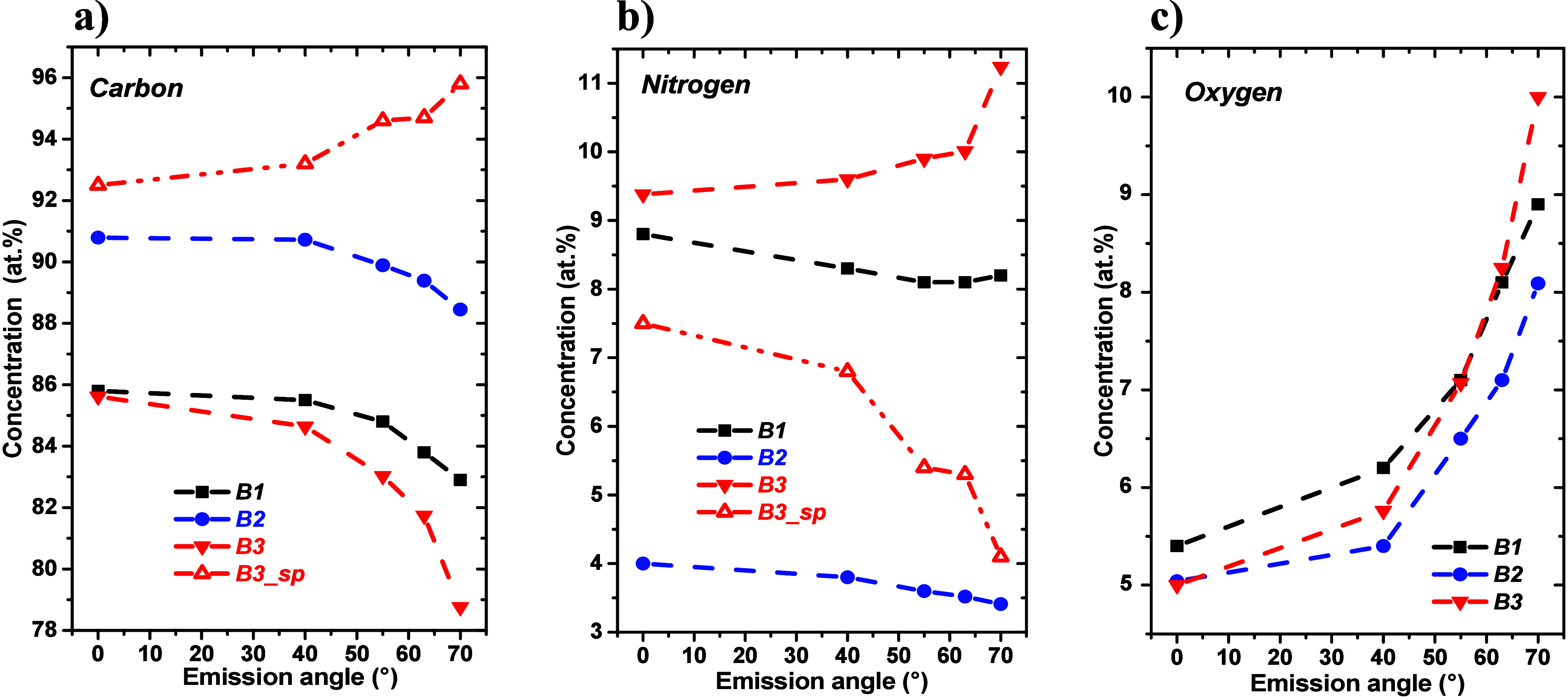
Photoelectron emission angle-dependent relative atomic fractions of (a) carbon, (b) nitrogen, and (c) oxygen evaluated from the spectra recorded for air-exposed surfaces of the DLC:N samples (B1–B3). Data for the sputter-cleaned surface, B3-sp, are added for comparison.

The angular dependencies of the apparent concentrations calculated from C 1s, N 1s, and O 1s peak areas recorded for the air-exposed surfaces of the analyzed samples are shown in Figure 3a–c, respectively.

The shape of the angular dependencies of carbon apparent concentrations recorded for air-exposed DLC:N surfaces, shown in Figure 3a, indicates that the carbon is somewhat reduced at the top surface at the expense of nitrogen and oxygen. The opposite behavior is observed only for the sputter-cleaned surface caused by the suppression of oxygen and nitrogen. The dependencies in Figure 3c clearly indicate that oxygen-bearing species are located at the surface of the samples, as expected from their air exposition and from the outcome of sputter cleaning. Angular dependencies of nitrogen concentrations appear to be more complicated to guess the in-depth distribution.

**Figure 4:**
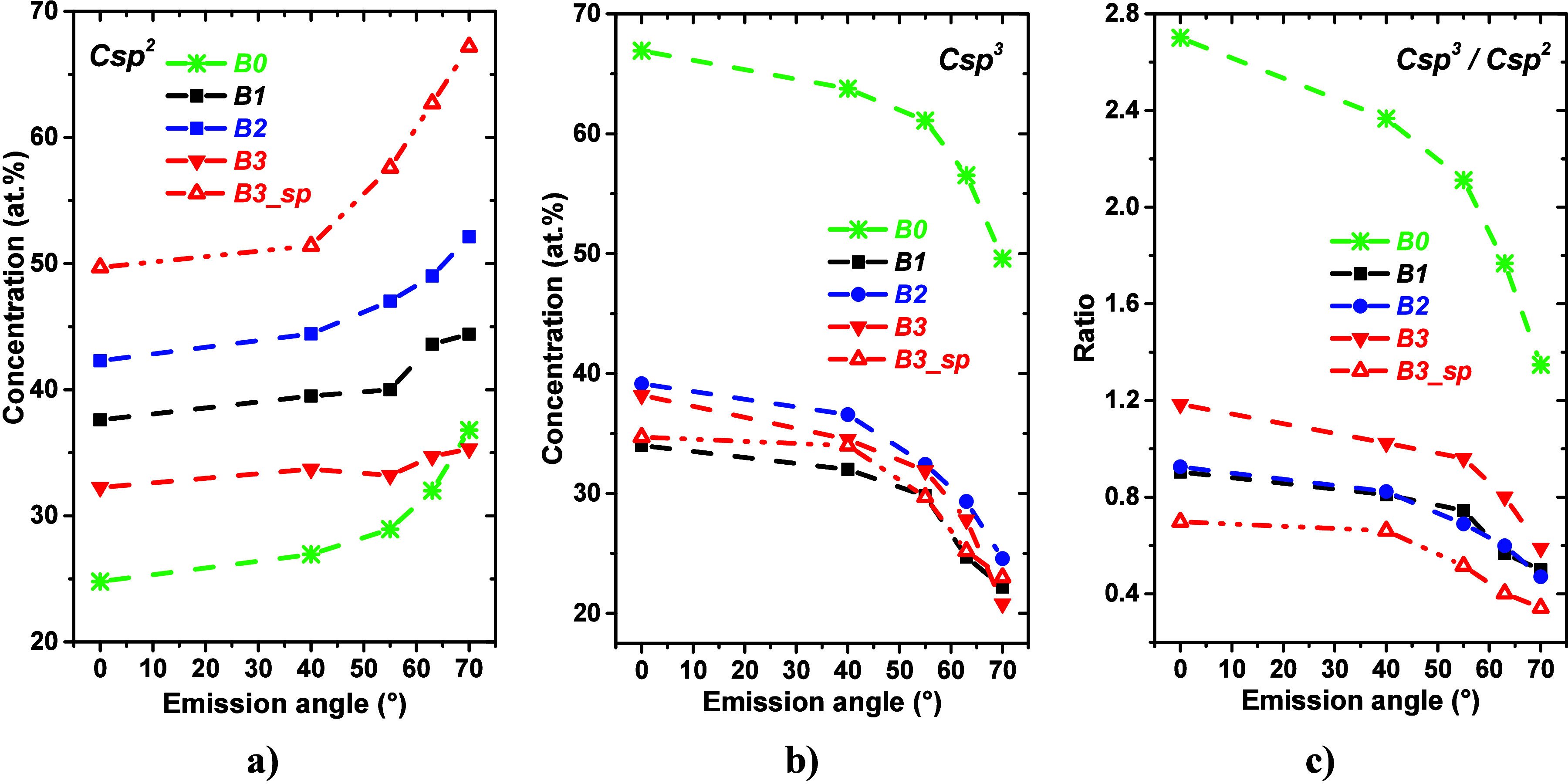
Photoelectron emission angle-dependent relative atomic fractions of (a) C sp^2^ and (b) C sp^3^ and (c) the C sp^3^/C sp^2^ ratio evaluated from the C 1s spectra recorded for the air-exposed DLC, DLC:N, and DLC:N sputter-cleaned (B3-sp) samples.

The shape of angular dependencies of C sp^3^, C sp^2^, and the C sp^3^/C sp^2^ ratio, shown in Figure 4, strongly indicates that the surface of the analyzed samples is enriched by C sp^2^ hybridization of carbon atoms.

Qualitatively, we conclude that the surfaces of the samples are enriched by oxygen-bearing species and by carbon atoms in sp^2^ hybridization, in agreement with the results of the surface composition and photoelectron spectral shape analysis. The C sp^3^ contribution dominates in a deeper subsurface region. It is worth noting that there is a significant decrease in C sp^3^ values on going from the DLC to DLC:N samples induced by the nitrogen insertion.

### Nondestructive Depth Profile Reconstructions

Photoelectron intensities depend also on the mass density in the near surface regions of the samples determined by the ID. Mass density values near the surfaces of the solids usually differ from their bulk value. To obtain the plausible in-depth distribution of elements using the MEM approach, it is required to know and to input the surface-related mass density values into the calculation.

### Surface-Related Mass Density

In the literature,^33−38^ there are measurements of macroscopic (averaged through the film thickness) mass densities of DLC:N films proving a decrease in density with an increase in nitrogen content. However, published surface-related mass density values for DLC:N films are scarce, presenting usually one density value or a density range.^27,39,40^

**Figure 5:**
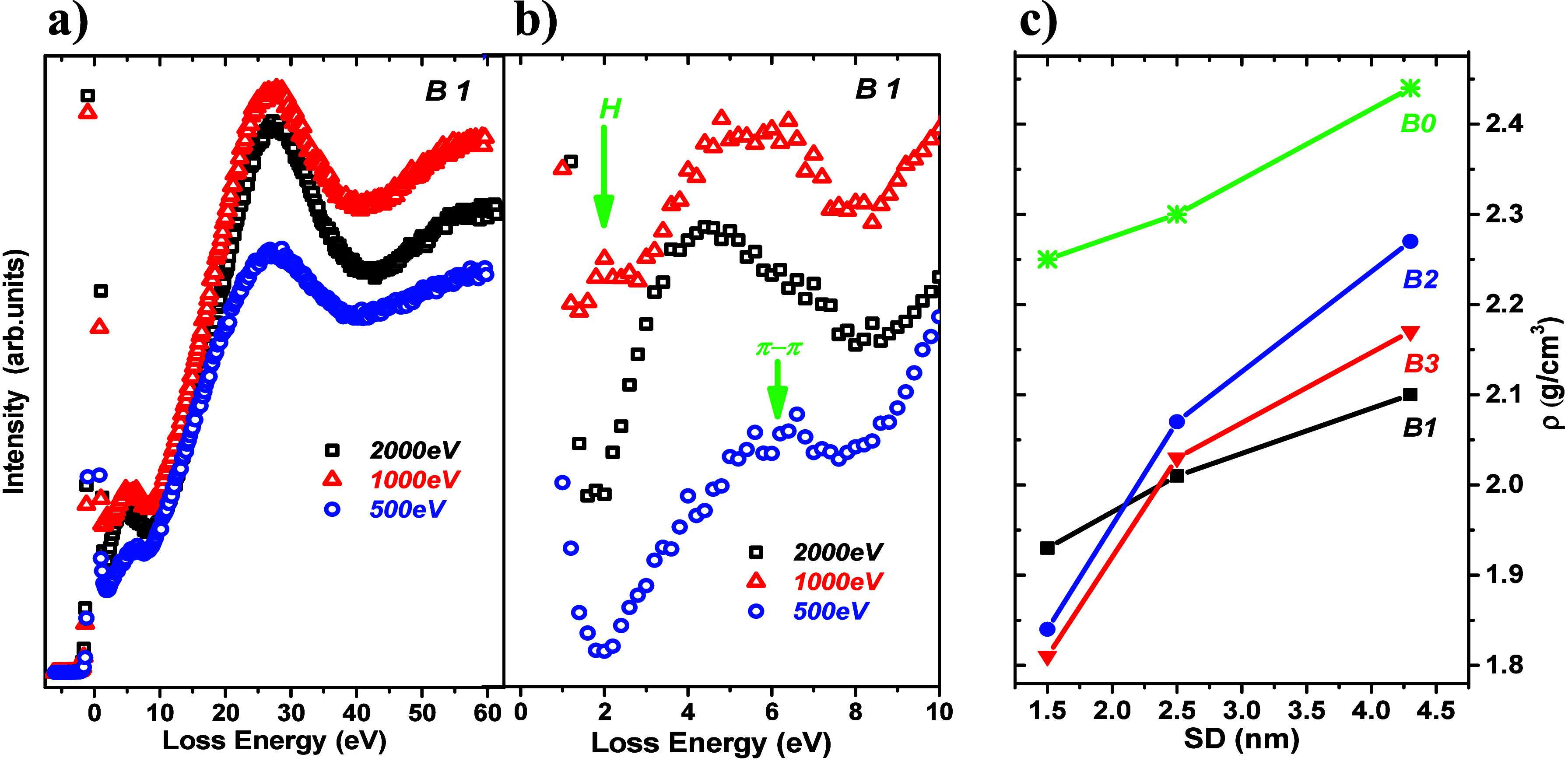
(a) Representative REELS spectra recorded for sample B1 at the indicated primary electron beam energies. The spectra, normalized to the intensity of the elastic peak, consist of π–π (2–7 eV) and π–σ (10–40 eV) transitions. (b) Expanded π–π region. The arrow indicates a tiny peak induced by hydrogen.^2^ (c) Resulting mass density values as a function of depth.

The surface-related and depth-dependent mass density values determined from the low-electron energy loss spectra excited at various primary beam electron energies are shown in panels a and b of Figure 5.

All of the spectra, normalized to the intensity of the elastic peaks, are characterized by two broad plasmon features due to collective excitations of π and π+σ electrons. The expanded π region, shown in Figure 5b, is characterized by wide blurred peaks with maxima shifted toward low loss energy with an increase in primary beam electron energy. The π loss peaks are composed of two subpeaks, discussed in ref (41), and explained by the modification of the π system because of the formation of nonplanar, nanometer-sized graphitic planes. In addition, there is a subtle spectral signal located at a loss energy of ∼2 eV, noticeable only for an electron primary beam energy of 1000 eV, ascribed to a recoil shift from hydrogen atoms likely bonded to oxygen as an -OH group.^2,42^ Upon examination of the spectra induced at electron energies of 500 and 2000 eV, the recoil shift expected at ∼1.9 and ∼3.8 eV^2^ is overlaid by the spectral signal from the elastic peak and the π loss feature, respectively. The mass density values derived from the REELS spectra (specifically from the position of the π+σ plasmon) are evaluated within the free electron model described in previous works.^14,39,43^ The resulting mass density values, averaged within the corresponding sampling depth of the REELS electrons, SD (defined by eq S2), are shown in Figure 5c, and the numerical data are summarized in Table S1. Generally, the SD < ID considering the same electron energy and the same material.^44,45^ Clearly, the density increases with the SD and with the depth for all samples, unlike the depth-independent surface-related density of highly oriented pyrolytic graphite (HOPG) (Table S1).

For the DLC:N films, the density ranges from ∼1.8 to ∼2.3 g/cm^3^, while for the nitrogen free DLC film, it ranges from 2.0 to 2.4 g/cm^3^. Therefore, the DLC:N films are less dense than the nitrogen free film. This is due to the introduction of nitrogen atoms, a lower content of C sp^3^-hybridized carbon atoms, and the formation of point vacancies in nitrogen ion-irradiated carbon films.^46^

### Application of the MEM Approach

In this calculation, we divide the near surface region of each sample into 12 parallel layers, which are 0.3 nm thick near the surface and 0.5 nm thick in a deeper region. The depth-dependent mass density of each layer is estimated from the data summarized in Table S1. An exponential form of the electron emission depth distribution function is assumed. IMFP values for C 1s, O 1s, and N 1s photoelectrons moving through graphite, 2.2, 1.8, and 1.9 nm, respectively, are taken from ref (45). As a preview model, we assume homogeneous in-depth distributions of the carbon, oxygen, and nitrogen atoms and bonding states that are resolved. Angle-resolved apparent concentrations of C sp^2^, C sp^3^, C≡N, N, C–O+C=O, and O evaluated from the respective peak areas, summarized in Tables S2–S6, are used as the MEM input data. To accelerate the calculation, only the dominating C≡N contribution is considered, and the sum of the C–O and C=O contributions is added up. Before profile reconstruction, the Laplace transform of the compositional depth profile is calculated from the angle-dependent apparent concentrations to test the consistency of the measured data with the layer model.^12^

**Figure 6:**
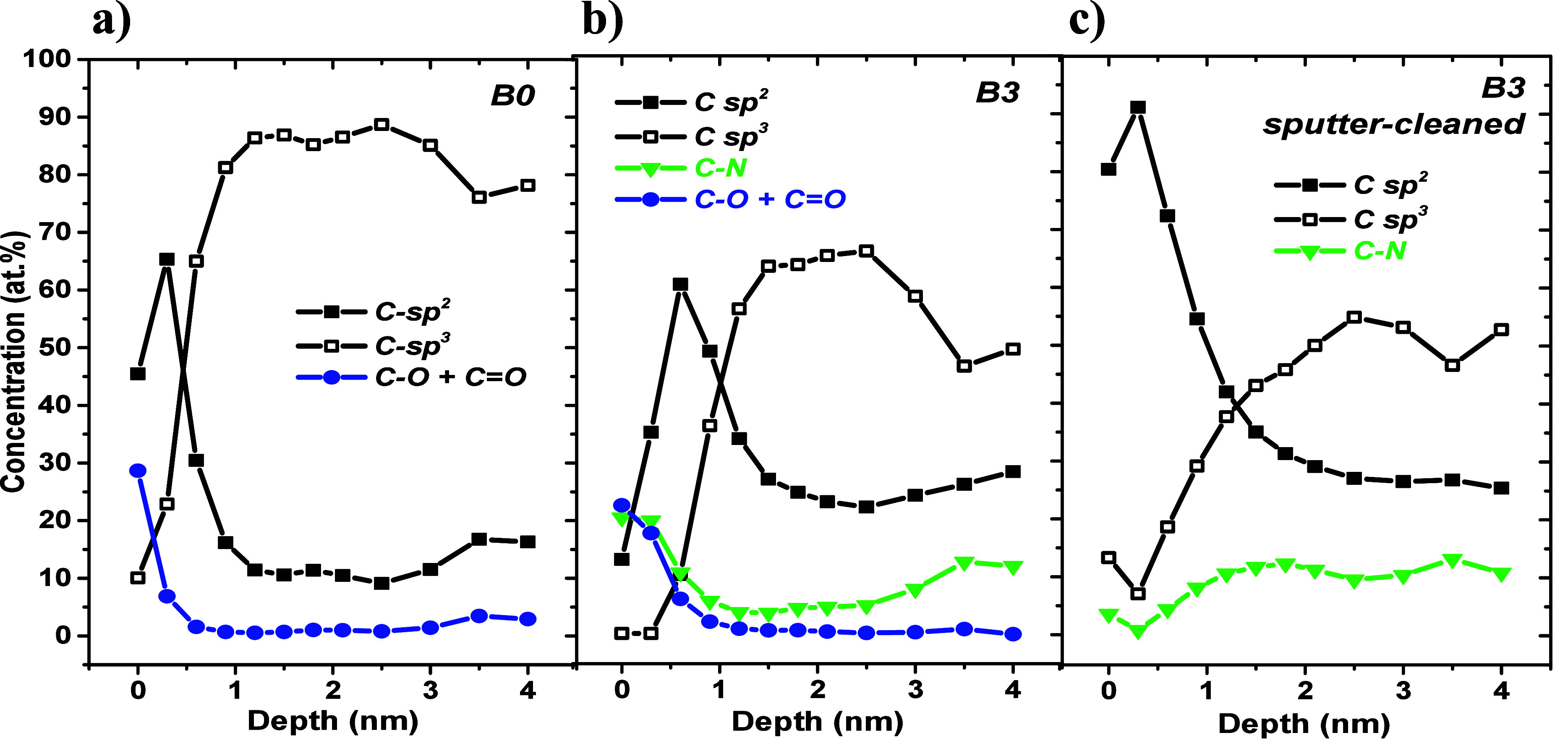
Concentration depth profiles (relative atomic fractions) of (a) air-exposed nitrogen free DLC, (b) air-exposed DLC:N, and (c) sputter-cleaned DLC:N sample surfaces.

**Figure 7:**
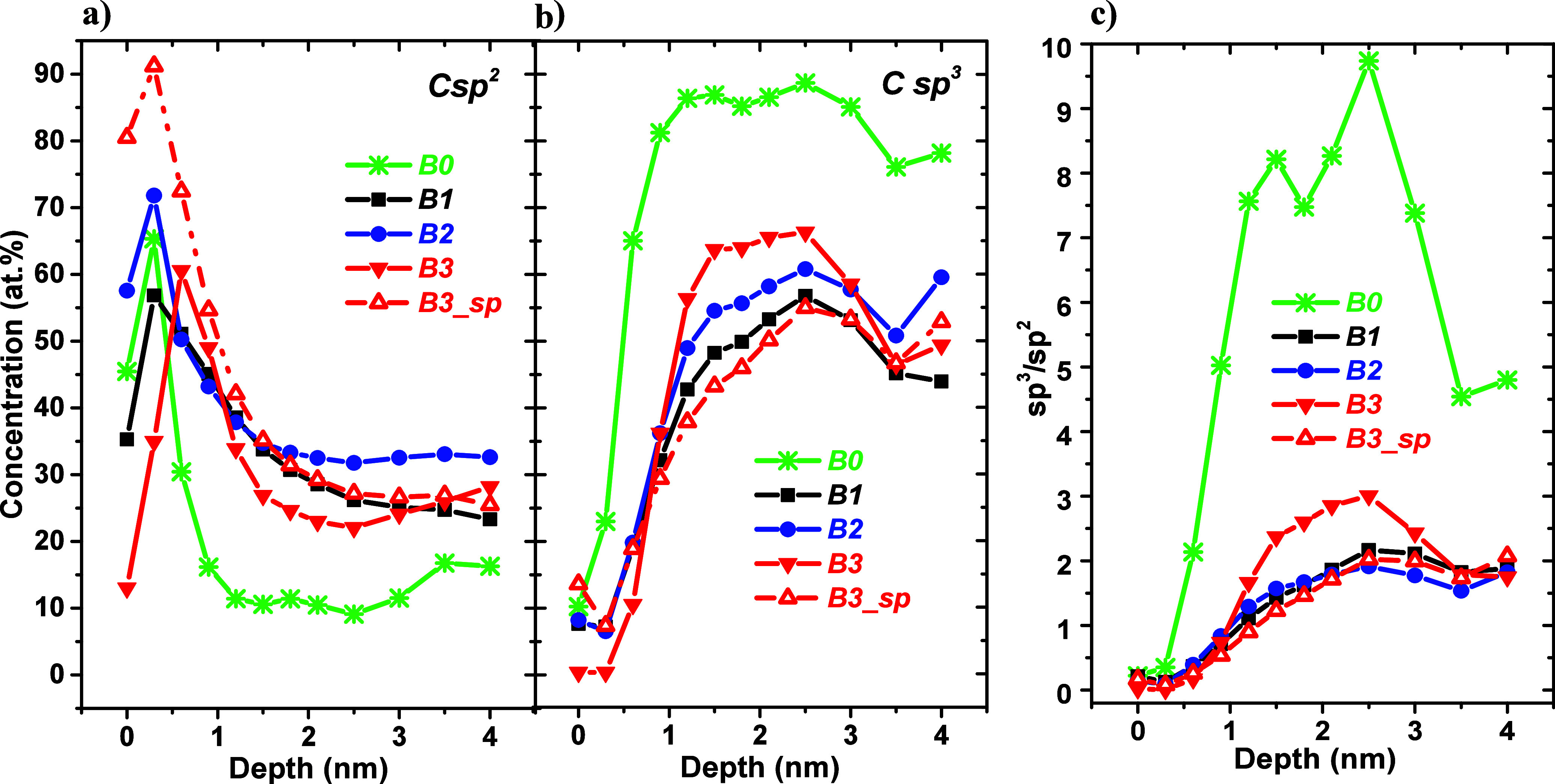
Concentration depth profiles (relative atomic fractions) of (a) C sp^2^ and (b) C(sp^3^b) contributions and (c) C sp^3^/C sp^2^ ratios.

**Figure 8:**
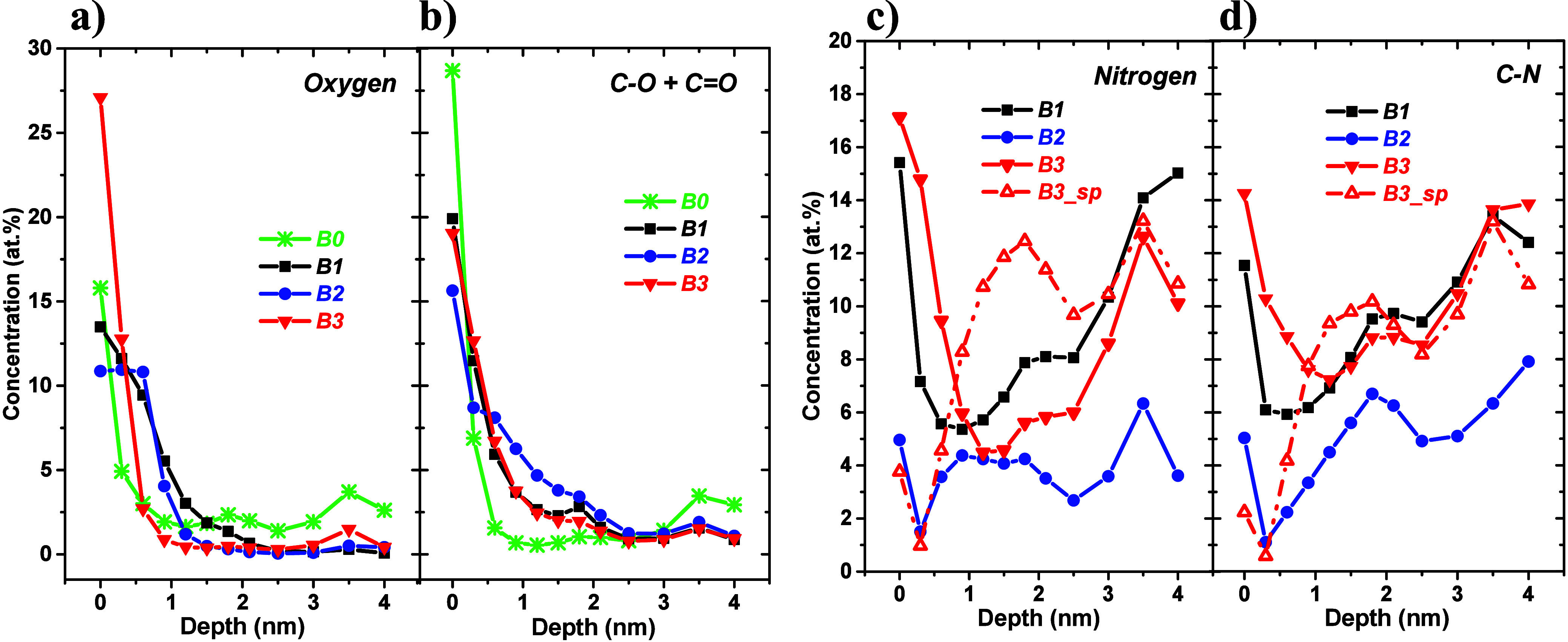
Concentration depth profiles (relative atomic fractions) of oxygen evaluated from the (a) O 1s lines, (b) carbon–oxygen (C–O+C=O) bonds evaluated from peak fits of the C 1s lines, (c) nitrogen evaluated from the N 1s lines, and (d) carbon–nitrogen bonds evaluated from peak fits of the C 1s lines.

The results of the depth profiles are shown in Figures 6–8. Figure 6 shows the concentration depth profiles of the air exposed surface of nitrogen-free DLC (a), air-exposed DLC:N (b), and sputtered cleaned DLC:N (c) samples.

The top surfaces of all of the samples are enriched by oxygen-bearing species and C sp^2^ bonds, and the DLC:N sample is also enriched with nitrogen (Figure 6b). Importantly, the C sp^2^ dominates in surface regions of ∼1 nm. For the nitrogen free DLC sample, C sp^2^ surface enrichment is expected. A similar effect has been observed independently by using spatially resolved electron loss spectroscopy, explained by the growth process,^47^ by comparison of C 1s and C KLL spectra,^48^ and by our previous works.^14,16^ DLC:N sample B3 was sputtered for a prolonged time of 60 min to remove even traces of oxygen; then, ARXPS spectra were recorded, and in-depth distributions were calculated (Figure 6c). One can expect that the near surface C sp^2^ peak is suppressed due to sputtering together with surface contamination. However, the C sp^2^ peak remains in existence and even increases. Note that the C sp^2^ maximum value shown in Figure 6b increases by only ∼10% if O and C–O+C=O are not considered. Apparently, sputter cleaning of DLC:N surfaces by Ar ion clusters contributes to the structural conversion of C sp^3^ to C sp^2^, even under an extremely low mean energy per Ar atom in Ar clusters. It is worth noting that the shapes of in-depth distributions for air-exposed and sputter-cleaned DLC:N samples are similar. This indicates that sputter cleaning modifies the surface region of 1 nm.

The in-depth distributions of C sp^2^ and C sp^3^ contributions and their ratios are shown in Figure 7.

The shapes of the profiles in Figure 7 are similar for all of the analyzed samples. However, there is a difference in concentration between the DLC and DLC:N samples. The origin of nitrogen-driven C sp^3^–C sp^2^ transformation has been investigated using *ab initio* Hartree–Fock density functional and semiempirical calculations on model clusters.^49^ These calculations suggest that with >12% nitrogen in DLC:N films there are thermodynamic and kinetic preferences for sp^2^- versus sp^3^-bonded structures. Kohler et al.^50^ have found that it is also valid for lower nitrogen contents. The in-depth distributions of oxygen- and nitrogen-bearing species are shown in Figure 8.

Panels a and b of Figure 8 clearly show that oxygen-bearing species are located at the top surfaces following an abrupt decrease within ∼1 nm of the profiles. Nitrogen distributions (Figure 8c,d; air-exposed DLC:N samples) are characterized by surface enrichment, followed by a depleted region and an expansion/saturation. The top surface of the sputter-cleaned DLC:N sample appears to be almost depleted of nitrogen atoms. The profiles seem to be slightly “wavy”; the origin of this feature is not completely clear at present. We believe that it is related to the dynamic character of the ending of DLC:N film growth that can influence element distributions in a surface region of the samples. This idea is supported by the pronounced surface segregation of nitrogen, documented by the results presented here (see Figures 1, 6, and 8 and published datain refs (5−7)) followed by a nitrogen-depleted subsurface region. Moreover, the nitrogen in-depth distributions behave approximately as a mirror of the C sp^3^ ones, confirming the nitrogen-induced C sp^3^–C sp^2^ transformation.

Evidently, we can conclude that qualitative and quantitative results are quite consistent. In particular, (i) oxygen-bearing species are located mainly at the surface of the samples, (ii) C sp^2^ bond fractions peak near the surfaces of the samples, and (iii) C sp^3^ bond fractions are mainly located beyond the surface in a deeper region of the samples.

Recently, ARXPS with the MEM has been helpful in revealing the in-depth distributions of carbon atom hybridizations in undoped DLC films,^14^ in understanding the Ge-induced cytotoxicity of Ge-doped DLC films,^51^ and in understanding the surface chemistry of Ca-doped DLC films,^16^ where we have observed the accumulation of calcium carbonate, CaCO_3_, at the surface, while the calcium oxide, CaO, is in a deeper subsurface region. In addition, we have found structural conversion of the C sp^2^-rich top surface observed in the undoped DLC layer^14^ to the C sp^3^-rich surface and conversion in the opposite direction, which occurred in a subsurface region.

The influence of surface roughness on the measured ARXPS spectral intensities is rather complicated. The photoelectron intensity measured from rough surfaces is mainly influenced by the true emission angles (the difference between nanoscopic/microscopic and macroscopic signal electron emission geometry) and electron and X-ray shadowing. We have developed a semiempirical method that makes it possible to include real surface roughness in calculations of surface composition and overlayer thickness.^52^ We have also shown that the currently used root-mean-square (rms) of heights does not work in photoemission, but the spread of a local area distribution of slopes can describe properly the influence of surface roughness on the spectra. In addition, the error induced by the random surface corrugation can be acceptably low for the spread of a local area distribution of slopes of <35°. In such a case, roughness features are rounded. For spiky-like roughness features, it is necessary to use an exacting way: to map the roughness of each analyzed sample surface by AFM, to model the roughness, and to calculate surface composition and/or overlayer thickness.

Extension of the method to carbon nanostructures is difficult, but not impossible. Application of ARXPS with the MEM is mostly reduced to the analysis of supports due to several limitations. Chiefly, the analyzed surface area should be amorphous or polycrystalline, homogeneous in composition (areal composition homogeneity), and ideally flat.^17^ As shown above, however, the problem with surface roughness can be neglected or solved by using the semiempirical method. Indeed, ARXPS with the MEM can be useful for revealing the composition and bonding states of atoms at the top surface and just below the surface. Therefore, the method can be useful for analyzing the surface composition and bonding in catalytic structures, in biomedical applications, and generally in analysis of carbon-based film surfaces upon interaction with gaseous or liquid surroundings.

## Conclusion

Determining surface and near surface composition and bonding quantitatively is a complicated task. To see beneath the surface, we applied angle-resolved X-ray-induced photoelectron spectroscopy to record the C 1s, O 1s, and N 1s spectra under various electron emission angles, evaluated by the MEM approach, i.e., to reveal in-depth distributions of elements and resolved bonding states at and just below the analyzed surfaces nondestructively. The results show that the in-depth distributions and mass density vary significantly from the top surface to a shallow subsurface region.

The surfaces of air-exposed DLC:N films are enriched with oxygen, nitrogen, and carbon atoms in C sp^2^ bonds. Moreover, hydrogen is identified in the REELS spectra. Sputter cleaning using an Ar ion cluster beam successfully removes adventitious carbon and oxygen-bearing species and suppresses nitrogen content at the top surface, whereas the spectral signal from Ar atoms is absent.

The C sp^2^ distribution forms a peak just beneath (within ∼1 nm) the air-exposed and sputter-cleaned surfaces. This peak is even enhanced in concentration due to the sputter-induced C sp^3^–C sp^2^ structural transformation. As a consequence, DLC and DLC:N layers with high mean C sp^3^/C sp^2^ ratios possess surfaces with carbon atoms in predominant sp^2^ hybridization.

In a deeper region, the C sp^3^ content increases substantially when going through a maximum. Similarly, the sp^3^/sp^2^ ratio goes to a maximum, then decreases, and tends to saturate. The nitrogen distribution behaves in an approximately mirror-like manner with respect to the sp^3^/sp^2^ ratio: it decreases from the surface value forming a minimum just below the surface, then increases, and tends to saturate.

It is worth noting that the calculated depth profiles are consistent with qualitative estimations of depth profiles, specifically with the results of the current apparent composition, photoelectron peak shapes, and photoelectron differential spectra and with angular dependencies of apparent concentrations of elements and resolved bonding states. With regard to the interaction between the DLC:N surfaces and surroundings, oxygen- and nitrogen-bearing species and, importantly, carbon atoms in sp^2^ bonds can be involved.

## Supporting Information Available

Survey photoelectron spectra, mass density evaluation, sampling depth calculation, and tables of apparent concentrations of elements and resolved bonding states (PDF)
