# Supplementary material for: Amorphous Carbon Nitride Films: Surface and Subsurface Composition and Bonding
Source: Langmuir. 2024 Aug 30;40(37):19538–47. doi: 10.1021/acs.langmuir.4c02007 (PMC11411727; doi:10.1021/acs.langmuir.4c02007)
Supplement: Supplementary file 1 — la4c02007_si_001.pdf [file la4c02007_si_001.pdf]

Supplementary Materials

Amorphous carbon nitride films: Surface and sub-surface composition and bonding

Josef Zemek[a,*], Jana Houdkova[a], Petr Jiricek[a], Tomas Kocourek[a,b]

[a]Institute of Physics of the Czech Academy of Sciences, Na Slovance 2, 182 21 Prague 8, Czech Republic
[b]Czech Technical University in Prague, Faculty of Biomedical Engineering,

nam. Sitna 3105, 27201 Kladno, Czech Republic

* Corresponding author. E-mail address: zemek@fzu.cz (J. Zemek).

Contents

1. Survey photoelectron spectra

2. Mass density and sampling depth calculation

3. Tables of apparent concentrations of elements and resolved bonding states

Photoelectron spectra

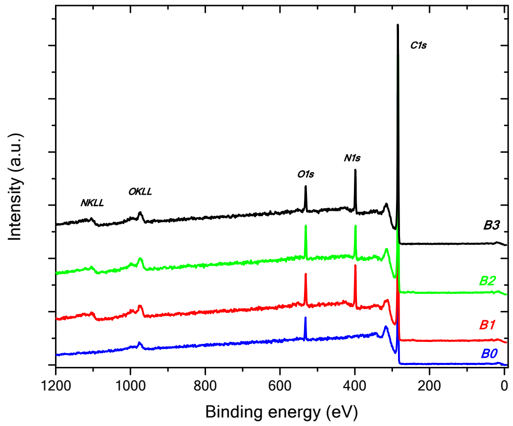

Figure S1: Typical photoelectron survey spectra recorded from the surfaces of the air-exposed DLC and DLC:N films

Mass density in near-surface regions of the samples derived from the low-loss electron spectra and sampling depths calculation

The low-loss electron spectra measured in the reflection mode (REELS) can be used to determine quantitatively the mass density ρ[1] from the bulk plasmon energy E_p by

$$\rho = \frac{\varepsilon_0 m\, M_c}{4h^2\, e^2 N_A} E_p^2 \qquad \text{(S1)}$$

Where ε_0 is the permittivity of free space, m is the effective electron mass, M_c is the molar mass of carbon, h is the Planck`s constant, $e$ is the electron charge, and $N_A$ is the Avogadro`s constant.

The mass density values are averaged within a sampling depth *(SD)*. The *SD* depends on the IMFP, experimental geometry, and the percentage of useful spectral intensity, *P*. We estimated the *SD* values from the formula derived for elastically backscattered electrons from graphite surface[2] for *P*=95% (95% of useful spectral signal originates from a surface region with a thickness equal to *SD,*).

$$SD = -\lambda \frac{sin\alpha_{in}\, sin\alpha_{out}}{sin\alpha_{in} + sin\alpha_{out}}\, ln\left(\frac{100-P}{100}\right) \qquad \text{(S2)}$$

α_{in} is the primary electron incidence angle, α_{out} is the backscattered electron emission angle. The *SD* values for the low-loss spectra excited by 500 eV, 1000 eV, and 2000 eV primary beam electron energy are 1.47 nm, 2.46 nm, and 4.28 nm, respectively.

Table S 1. Plasmon energy *Ep* evaluated from the low-loss spectra excited at 500 eV, 1000 eV, and 2000 eV primary electron beam energy and mass density ρ calculated from eq. (S1). As expected, the mass density values for HOPG are electron energy independent, while the values derived from the spectra of the DLC and CN_x films increase with electron energy.

Sample	Electron energy (eV)					
	2000		1000		500	
	E_p (eV)	ρ (g/cm³)	E_p (eV)	ρ (g/cm³)	E_p (eV)	ρ (g/cm³)
HOPG	27.1	2.20	27.2	2.23	26.9	2.18
B0	28.0	2.36	27.2	2.23	25.7	1.99
B1	26.5	2.10	25.9	2.01	25.4	1.93
B2	27.1	2.27	26.3	2.07	24.8	1.84
B3	26.9	2.17	26.0	2.03	24.6	1.81
SD (nm)	4.3		2.5		1.5	

Tables of apparent concentrations of elements and resolved bonding states

Table S2: Apparent concentrations of element and resolved chemical bonds of sample B0

α (°)	C sp²	C sp³	C-O+C=O	O
0	24.78	66.93	4.30	4.00
40	26.94	63.76	4.94	4.36
55	28.93	61.10	5.38	4.60
63	32.00	56.54	6.34	5.13
70	36.80	49.60	7.70	5.90

Table S3: Apparent concentrations of elements and resolved chemical bonds of sample B1

**α (°)**	**C sp^2**	**C sp^3**	**C-O+C=O**	**C≡N**	**O**	**N**
0	33.82	36.12	5.00	8.50	5.00	8.50
40	36.75	34.44	6.00	8.47	6.00	8.40
55	37.87	32.41	6.51	8.14	6.70	8.10
63	40.21	29.58	7.62	7.50	7.70	7.60
70	41.65	26.06	8.72	7.70	8.70	7.80

Table S4: Apparent concentrations of elements and resolved chemical bonds of sample B2

α (°)	***C sp^2***	***C sp^3***	***C-O+C=O***	***C≡N***	***O***	***N***
0	40.30	40.20	5.48	4.17	5.04	4.21
40	42.22	36.18	5.93	4.22	5.53	3.92
55	47.02	32.32	6.80	3.75	6.50	3.60
63	49.01	29.33	7.53	3.51	7.10	3.52
70	52.31	24.56	8.14	3.31	8.09	3.41

Table S5: Apparent concentrations of elements and resolved chemical bonds of sample B3

α (°)	***C sp²***	***C sp³***	***C-O+C=O***	***C≡N***	***O***	***N***
0	31.14	39.36	5.20	9.90	5.14	9.77
40	30.98	37.11	5.90	9.70	5.84	9.63
55	32.97	33.87	6.90	9.69	6.79	9.49
63	33.94	31.53	7.80	9.43	7.63	9.54
70	36.53	25.85	8.90	9.98	8.78	9.98

Table S6: Apparent concentrations of elements and resolved chemical bonds of the sputter-cleaned sample B3

α (°)	***C sp²***	***C sp³***	***C≡N***	***N***
0	49.2	33.7	9.1	7.5
40	51.4	34.0	7.8	6.8
55	57.6	29.7	7.3	5.4
63	62.7	25.2	6.8	5.3
70	67.2	23.0	5.6	4.1

References

1. Bosman, M.; Keast, V.J.; Watanabe, M.; McCulloch, D.G.; Shakerzadeh, M.; Teo, E.H.T.; at al. Quantitative, nanoscale mapping of sp^2 percentage and crystal orientation in carbon multilayers. *Carbon* **2009**, *47*, 94-101.
2. Jablonski, A.; Powell, C.J. Information depth for elastic-peak electron spectroscopy. *Surf. Sci.* **2004,** *551.* 106-124.
